# 689. Significant Improvement in Hand Hygiene Compliance and Decline in *Clostridioides difficile* Infections with Implementation of Infection Prevention Interventions

**DOI:** 10.1093/ofid/ofad500.751

**Published:** 2023-11-27

**Authors:** Ashlesha Kaushik, Kristen Beal, Sandeep Gupta

**Affiliations:** UnityPoint Health and University of Iowa Carver College of Medicine, Sioux City, Iowa; UnityPoint Health, Sioux City, Iowa; Pulmonary and Critical Care Medicine, Unity Point Health, Sioux City, Iowa

## Abstract

**Background:**

Per CDC, *C. difficile* is an urgent microbial threat. Despite use of CDC diagnostic criteria, 2-step testing protocol and antimicrobial stewardship interventions limiting broad spectrum antimicrobial use, we witnessed an increase in infections in 2022, therefore, additional interventions were implemented.

**Methods:**

A multifaceted QI intervention program to improve IPC (infection prevention and control) practices was implemented in July 2022 at a 160-bed tertiary care center serving the tristate area of Iowa, South Dakota and Nebraska. Interventions included: auditing and real time observations for health care personnel (HCP) hand hygiene compliance; monitoring soap/sanitizer usage; educational interventions including posters posted throughout the hospital emphasizing hand hygiene, *“A clean in and clean out”* approach and contact precautions reminders posted in patient rooms/nursing stations, weekly nursing huddles focused on nursing and ancillary staff education, provider counseling and continued coaching. Pre-intervention period (P1: 1/10/2021- 6/30/2022) was compared with Intervention period (P2: 07/01/2022-3/31/2023) for hand hygiene compliance and *C. difficile* infection rates.

**Results:**

Overall adequate hand hygiene compliance rates for all health care personnel (HCP) increased from 71% during P1 to 85% during P2 (P< 0.05). All categories of HCP showed a significant improvement in hand hygiene compliance from P1 to P2, with rates for physicians increasing from 74% to 92% (p< 0.05), nurses from 68% to 94% (p< 0.001); nurse practitioners/physician assistants from 75% to 90% (p< 0.05), ancillary staff (chaplain, medical technicians, social workers) from 60% to 89% (p< 0.01)]. Hand hygiene product use increased significantly and soap usage alone increased from 45% to 75% in P2 (p< 0.05). *C. difficile* infections declined significantly from a mean of 1.9 infections per 1000 patient days during P1 to 0.5 infections per 1000 patient days during P2 (p< 0.001); SIR (Standardized infection ratio) decreased from 1.14 during P1 to 0.4 during P2 (p< 0.01). No *C. difficile* related mortality was observed.

**Conclusion:**

With implementation of multifaceted IPC interventions, significantly improved hand hygiene rates and a significant decline in *C. difficile* infections were observed.

**Disclosures:**

**All Authors**: No reported disclosures

